# Diurnal Variations in High Time-Resolved Molecular Distributions and Formation Mechanisms of Biogenic Secondary Organic Aerosols at Mt. Huang, East China

**DOI:** 10.3390/molecules28165939

**Published:** 2023-08-08

**Authors:** Yuanyuan Li, Zhanfang Hou, Yachen Wang, Tonglin Huang, Yanhui Wang, Jiangkai Ma, Xiuna Chen, Aimei Chen, Min Chen, Xiaoting Zhang, Jingjing Meng

**Affiliations:** 1 School of Geography and Environment, Liaocheng University, Liaocheng 252000, China; liyuanyuan1428@163.com (Y.L.); wangyachen0809@163.com (Y.W.); htl19980929@163.com (T.H.); wyh132719@163.com (Y.W.); mjk19990324@163.com (J.M.); chenmin0635@163.com (M.C.); zyzxt929@163.com (X.Z.); mengjingjing@lcu.edu.cn (J.M.); 2 State Key Laboratory of Loess and Quaternary Geology, Key Laboratory of Aerosol Chemistry and Physics, Institute of Earth Environment, Chinese Academy of Sciences, Xi’an 710075, China; 3 Institute of Huanghe Studies, Liaocheng University, Liaocheng 252000, China; 4 Liaocheng Ecological Environment Monitoring Center of Shandong Province, Liaocheng 252000, China; chenxiuna@163.com; 5 Municipal Bureau of Ecological Environment of Liaocheng, Liaocheng 252000, China; chenaimei@126.com

**Keywords:** biogenic secondary organic aerosol (BSOA), diurnal variations, mountain-valley breezes, aqueous-phase oxidation, Mt. Huang

## Abstract

The molecular characteristics and formation mechanism of biogenic secondary organic aerosols (BSOAs) in the forested atmosphere are poorly known. Here, we report the temporal variations in and formation processes of BSOA tracers derived from isoprene, monoterpenes, and *β* caryophyllene in PM_2.5_ samples collected at the foot of Mt. Huang (483 m a. s. l) in East China during the summer of 2019 with a 3 h time resolution. The concentrations of nearly all of the detected species, including organic carbon (OC), elemental carbon (EC), levoglucosan, and SIA (sum of SO_4_^2−^, NO_3_^−^, and NH_4_^+^), were higher at night (19:00–7:00 of the next day) than in the daytime (7:00–19:00). In addition, air pollutants that accumulated by the dynamic transport of the mountain breeze at night were also a crucial reason for the higher BSOA tracers. Most of the BSOA tracers exhibited higher concentrations at night than in the daytime and peaked at 1:00 to 4:00 or 4:00 to 7:00. Those BSOA tracers presented strong correlations with O_3_ in the daytime rather than at night, indicating that BSOAs in the daytime were primarily derived from the photo-oxidation of BVOCs with O_3_. The close correlations of BSOA tracers with SO_4_^2−^ and particle acidity (pH_is_) suggest that BSOAs were primarily derived from the acid-catalyzed aqueous-phase oxidation. Considering the higher relative humidity and LWC concentration at night, the promoted aqueous oxidation was the essential reason for the higher concentrations of BSOA tracers at night. Moreover, levoglucosan exhibited a robust correlation with BSOA tracers, especially *β*-caryophyllinic acid, suggesting that biomass burning from long-distance transport exerted a significant impact on BSOA formation. Based on a tracer-based method, the estimated concentrations of secondary organic carbon (SOC) derived from isoprene, monoterpenes, and *β* caryophyllene at night (0.90 ± 0.57 µgC m^−3^) were higher than those (0.53 ± 0.34 µgC m^−3^) in the daytime, accounting for 14.5 ± 8.5% and 12.2 ± 5.0% of OC, respectively. Our results reveal that the BSOA formation at the foot of Mt. Huang was promoted by the mountain-valley breezes and anthropogenic pollutants from long-range transport.

## 1. Introduction

Secondary organic aerosols (SOAs) are important components of atmospheric fine particulate matter (PM_2.5_), which have adverse effects on global climate change, atmospheric visibility, cloud condensation, and human health [1,2,3]. The homogeneous and heterogeneous reactions of volatile organic compounds (VOCs) are essential in the formation of SOAs [4,5]. It is known that vegetation can release a large quantity of biogenic VOCs (BVOCs) into the atmosphere, including monoterpenes (15%), isoprene (50%), and sesquiterpenes (3%) [6,7]. BVOC emissions are estimated to be an order of magnitude higher than anthropogenic VOCs (AVOCs) on the global scale, contributing significantly to the increase in SOA concentration levels [8]. Therefore, biogenic secondary organic aerosols (BSOAs) have attracted much more attention in recent years and have become the frontier and hot topic of air pollution research. However, the formation and transformation mechanisms of BSOAs are still less well known to date.

Investigations on the characteristics of BSOA tracers derived from isoprene, monoterpene, and *β*-caryophyllene have been conducted in various environments, such as rural [9,10], urban [11], mountainous [12,13,14], and marine [15] regions. There have been relatively few studies on BSOAs in forested highland regions [16], especially with a high time resolution. Field observations about BSOAs at a high time resolution are helpful to understand the evolution processes, formation mechanisms, and controlling factors of BSOAs. Mochizuki et al. [17] pointed out that the photochemical oxidation of BVOCs such as α-pinene and isoprene with O_3_ was the essential source for BSOAs in the forest ecosystem with a 3 h time resolution. Li et al. [18] carried out an observation campaign with a 3 h interval at a rural site in the North China Plain and reported that regional biomass burning contributed significantly to the terpene-derived SOA production. A few studies demonstrated that mountain-valley breezes and anthropogenic pollutants (e.g., biomass burning) from long-distance transport played an important role in the BSOA formation at the summit of mountains [12,14,19,20]. However, there is little information about the temporal variations, evolution mechanisms, and determining factors of BSOAs in mountainous forested regions at a high time resolution, especially in East China.

Compared with the plain areas, mountains provide a unique environment for investigating BSOAs due to the stronger solar radiation and higher relative humidity in high-altitude areas [13]. Air pollution is still a primary environmental issue in East China, despite a continuous improvement in air quality due to multiple regulatory policies [21,22]. Mt. Huang, the highest mountain in Eastern China (1840 m, a. s. l), provides an ideal sampling site for investing the molecular characteristics and formation mechanisms of BSOAs in PM_2.5_ samples in the background region of Eastern China. To the best of our knowledge, research on the characteristics and evolution mechanisms of BSOAs at Mt. Huang has not been carried out. In this study, we conduct high time-resolution sampling with a 3 h interval and investigate the temporal variations in molecular characteristics, sources, and formation pathways of BSOA tracers derived from isoprene, monoterpene, and *β*-caryophyllene at Mt. Huang and then explore the effect of meteorological factors, aerosol aqueous properties (e.g., liquid water content (LWC) and particle acidity (pH_is_)), anthropogenic pollutants, and mountain-valley breezes on their formation processes in the forested atmosphere.

## 2. Results and Discussion

### 2.1. Concentrations and Major Chemical Compounds of PM_2.5_

**Figure 1:**
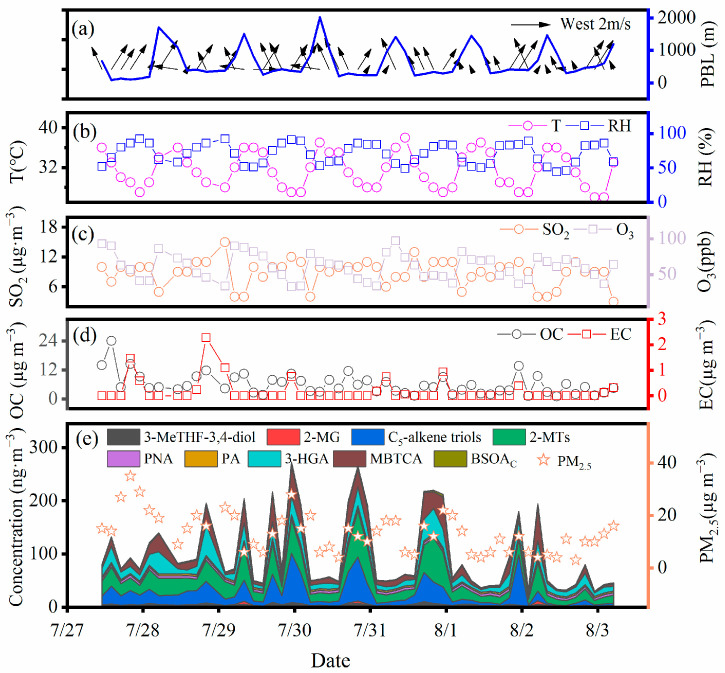
Temporal variations of (**a**) Wind speed, direction and PBL height, (**b**) Temperature and relative humidity, (**c**) SO_2_ and O_3_, (**d**) OC and EC and (**e**) Concentration of PM_2.5_ and BSOA tracers (2-MG: 2-methylglyceric acid; 2-MTs: 2-methyltetrols; PNA: *cis*-pinonic; PA: *cis*-pinic; 3-HGA: 3-hydroxyglutaric acid; MBTCA: 3-methyl-1,2,3-butanetricarboxylic acid; BSOAc: *β*-caryophyllenic acid).

The diurnal variations in PM_2.5_ concentrations, major chemical compounds (e.g., OC, EC, and SIA (sum of SO_4_^2−^, NO_3_^−^, and NH_4_^+^)), meteorological parameters, and gaseous pollutants are illustrated in Table 1 and Figure 1. The mean mass concentration of PM_2.5_ (3 h) was 13.3 ± 7.3 µg m^−3^ in the whole observation period, which was significantly lower than that at Mt. Tai (37 ± 16 µg m^−3^) in the summer [23] and in surrounding megacities (e.g., Hefei, Shanghai, Nanjing, and Hangzhou) (19.3 ± 2.3 µg m^−3^) of the YRD during the corresponding period of observation (https://www.aqistudy.cn/, accessed on 21 November 2022). According to the Chinese National Ambient Air Quality Standard (35 µg m^−3^), the air quality at Mt. Huang was good. Therefore, Mt. Huang can be considered as an ideal rural background site for studying pollution characteristics in East China. Interestingly, the concentration level of PM_2.5_ over Mt. Huang was almost equal to that at Mt. Wuyi (14 ± 7.8 µg m^−3^) in summer [12], representing a representative background level in the atmosphere of China.

The daytime (7:00–19:00) concentrations of both OC and EC were lower than those at night (19:00–7:00 of the next day), but the ratios of OC/EC were similar during the daytime and night-time (Table 1). Secondary organic carbon (SOC) was associated with various physical and chemical transformation processes of aerosols, which can be used to assess SOA pollution levels [24]. As shown in Table 1, the night-time concentration of SOC was 2.6 times higher than that in the day, indicating more SOA production from the aqueous oxidation at night. Levoglucosan is principally derived from the pyrolysis of cellulose and hemicellulose at temperatures higher than 300 °C, and thus it can be recognized as a significant tracer of biomass burning [25]. The average concentration of levoglucosan was as low as 2.4 ± 2.6 ng m^−3^, and the percentage of C from levoglucosan was 0.018 ± 0.016% in OC. Similar to the diurnal variations in PM_2.5_, OC, EC, and SOC concentrations, concentrations of SIA at night were also higher than those in the day, largely because of the dynamic transport of air pollutants via mountain breezes [26]. The difference in solar radiation leads to a large temperature difference between the peak and the foot of the mountain during the day and night, which in turn generates a difference in atmospheric pressure and forms the mountain-valley winds. The mountain breeze prevailed at night, and atmospheric pollutants were more likely to accumulate in the valley. In addition, the higher humidity (75.6 ± 12.2%) at night and larger daily range of temperature (10 °C) (Table 1, Figure S1) were conducive to the gas-to-particle transformation of organic compounds [27]. Similarly, SO_2_ and NO_2_ at night-time also presented higher concentrations than those in daytime (Table 1). The LWC of aerosol is controlled by both SIA concentration and RH [9]. Considering the higher SIA concentration and RH at night, LWC presented a higher concentration at night than that in the day (Table 1). Conversely, pH_is_ showed a higher value in the day than that at night (Table 1), indicating that aerosols at night were more acidic.

### 2.2. Diurnal Variations in BSOA Tracers

A total of 13 BSOA tracers were identified in the PM_2.5_ samples, which can be classified into three categories: SOA_I_, SOA_M_, and SOA_C_ (Table 2). The total concentration of BSOA tracers at night was 1.7 times higher than that in the day. SOA_I_ tracers were the most abundant species, accounting for 56.1% and 61.5% of the total determined BSOA tracers during the daytime and night-time, respectively, followed by SOA_M_ tracers (41.8% and 36.6%) and SOA_C_ tracers (2.1% and 1.9%).

**Figure 2:**
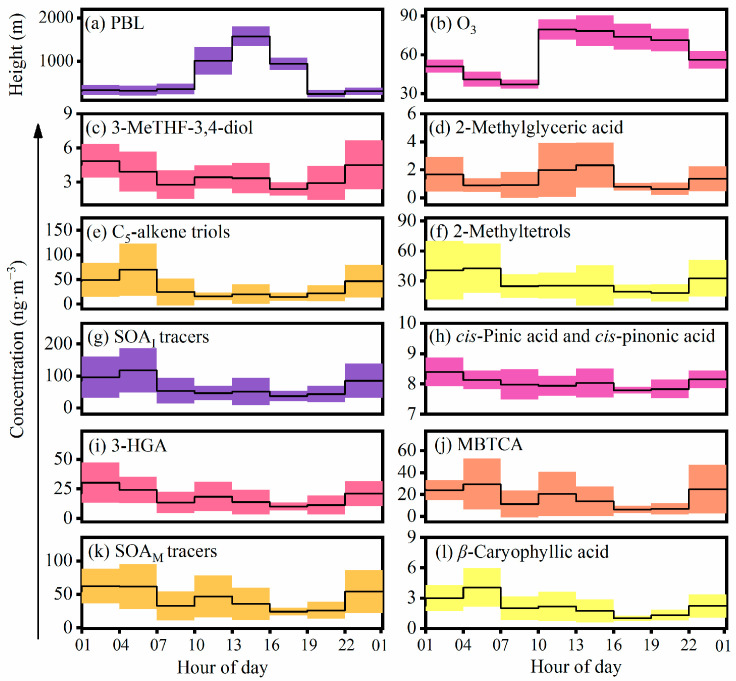
Diurnal variations in the detected BSOA tracers, planetary boundary layer (PBL) height, and O_3_.

**Figure 3:**
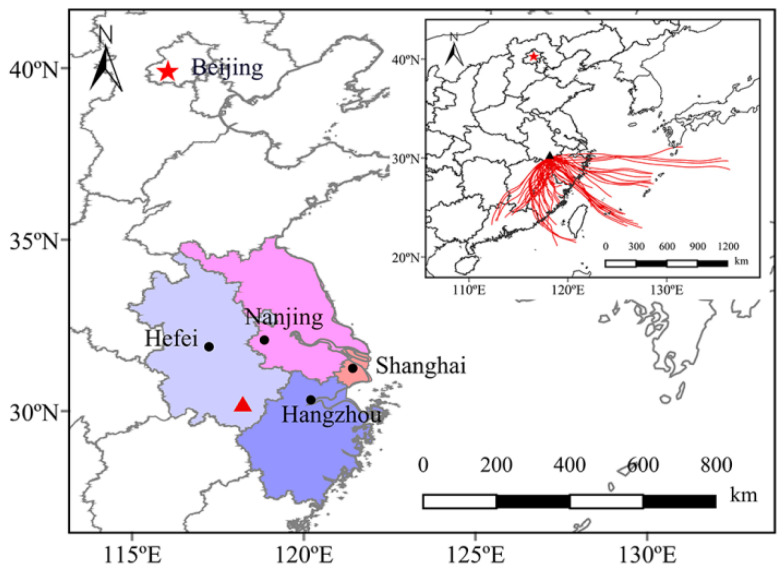
Map showing the location of Mt. Huang and the 48 h backward trajectories of air mass arrivals at Mt. Huang in the whole observation period. The triangle represents the sampling point.

Diurnal variations (3 h interval) in the BSOA tracers, O_3_ concentrations, and the PBL are shown in Figure 2. Nearly all of the BSOA tracers, except for 2-methylglyceric acid, presented higher concentrations at night than during the day, and culminated at 1:00 to 4:00 or 4:00 to 7:00. RH, LWC, SO_4_^2−^, and NO_3_^−^ also peaked during the above time periods (Figure S1). Higher RH and LWC can reduce the viscosity of the aerosol particles and enhance the hygroscopicity, which in turn promotes the generation of BSOAs [28]. In addition, according to the backward trajectory (Figure 3), pollutants from long-distance transport were transported and accumulated at the foot of the mountain with the effect of the prevailing mountain breeze. According to Table 2, the ratio of BSOA tracers to OC was greater at night than in the day, indicating that the PBL height was not a major reason for the increase in BSOA tracers at night. The daytime concentrations of SOA_I_ and SOA_M_ tracers reached the highest values at 10:00 to 13:00 or 13:00 to 16:00, which was consistent with the variation inn O_3_, largely because of the enhanced photochemical oxidation driven by O_3_ [11].

#### 2.2.1. Isoprene SOA Tracers

The night-time concentration (87.0 ± 61.2 ng m^−3^) of SOA_I_ tracers was 1.9 times higher than that (46.6 ± 32.1 ng m^−3^) in the day (Table 2). The molecular distribution of SOA_I_ tracers was characterized by the dominance of 2-methyltetrols during the day and C_5_-alkene triols during the night, and 3-MeTHF-3,4-diol and 2-methylglyceric acid were the two lowest species during the daytime and night-time (Figure 2).

**Figure 4:**
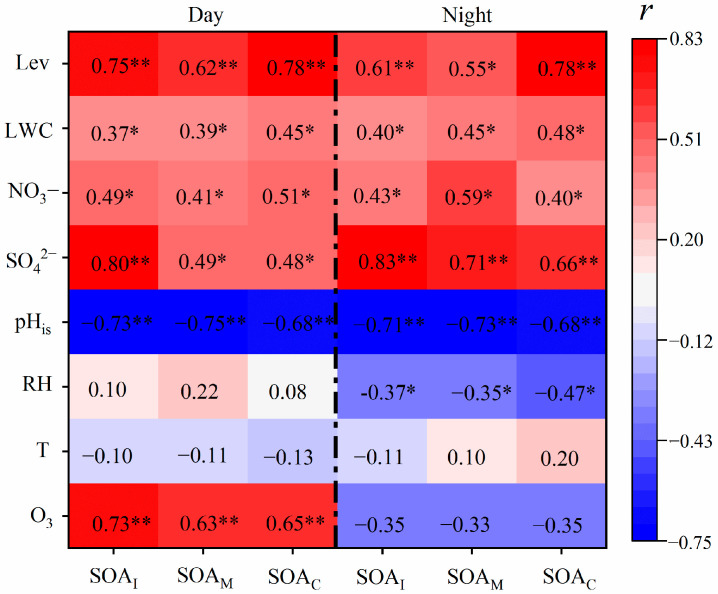
Correlation analysis (*r*) of SOA tracers with levoglucosan (Lev), liquid water content (LWC), NO_3_^−^, SO_4_^2−^, in situ particle pH (pH_is_), relative humidity (RH), temperature (T), and O_3_ during the daytime and nighttime, respectively. Correlation coefficients with an asterisk (*) indicate statistically significant relationships at 0.01 < *p* < 0.05, two asterisks (**) means highly significant relationships at 0.001 < *p* < 0.01, and no asterisk indicates not significant relationships at *p >* 0.05.

3-MeTHF-3,4-diols, 2-methyltetrols, and C_5_-alkene triols can be primarily produced from the reactive uptake of isoprene epoxydiols (IEPOX) formed by the oxidation of isoprene with RO_2_ and HO_2_·radicals in the absence of NO_x_ [11]. IEPOX can be partitioned from the gaseous phase into the aerosol phase and ultimately generate 3-MeTHF-3,4-diols and/or C_5_-alkene triols via acid-catalyzed intermolecular rearrangement reactions [27]. C_5_-alkene triols can be derived from the oxidation of 3-MeTHF-3,4-diol with acidic particles [29]. Therefore, C_5_-alkene triols presented a strong linear correlation with 3-MeTHF-3,4-diol during the day (*r* = 0.81, *p* < 0.01) and night (*r* = 0.87, *p* < 0.01). It is worth noting that the slope (ratio of C_5_-alkene triols to 3-MeTHF-3,4-diol) of the regression line at night (16.6) was almost twice higher than that (8.9) during the daytime (Figure S2), indicating the stronger ring-opening reactions of the yield of C_5_-alkene triols from 3-MeTHF-3,4-diol at night [30]. The enhanced aqueous-phase oxidation of IEPOX derived from the oxidation of isoprene in the acidic/neutral particles at night was responsible for the higher night-time concentration of 2-methyltetrols [31,32]. Different from other detected BSOA tracers, the concentration of 2-methylglyceric acid in the day was 1.2 times higher than that at night (Table 2), which might be ascribed to the lower relative humidity conditions in the day. Field measurements and chamber studies pointed out that 2-methylglyceric acid is mainly derived from the oxidation of isoprene under high NO_x_ conditions and is facilitated into the aerosol phase under the less humid environment [13,32,33]. As shown in Figure 4, there were robust correlations between SOA_I_ tracers and SO_4_^2−^ during the daytime (*r* = 0.80, *p* < 0.01) and night-time (*r* = 0.83, *p* < 0.01), suggesting that the aqueous-phase chemistry reaction was an important pathway for the formation of SOA_I_ tracers in the atmosphere.

#### 2.2.2. Monoterpene SOA Tracers

The concentration of total SOA_M_ tracers at night was 1.5 times higher than that in the day. 3-HGA was the most abundant SOA_M_ tracer, which accounted for 40.0% and 42.5% in the total SOA_M_ tracers during the day and night, respectively, followed by MBTCA (37.2% versus 41.8%), *cis*-pinonic acid (13.7% versus 9.3%), and *cis*-pinic acid (9.1% versus 6.4%). *Cis*-Pinonic acid and *cis*-pinic acid are the first-generation products of SOA_M_ tracers, which can be further photodegraded by HO· radicals to form MBTCA [34]. Thus, the P/M ratio (sum of *cis*-pinonic acid and *cis*-pinic acid to MTBCA) can be proposed as an essential tracer for the aging degree of SOA_M_ [35]. The higher P/M ratio reflects the fresher organic aerosols [35]. A chamber study showed that the P/M ratio varied from 1.51 to 3.21 in fresh *α*-pinene SOA samples [36]. In the aerosols of Mt. Huang, the average ratio (1.1 ± 0.6) of P/M in the daytime was 1.4 times higher than that (0.8 ± 0.6) at night, suggesting that SOA_M_ was more aged at night due to the enhanced aqueous oxidation. The lowest ratio (0.41) of P/M appeared at 4:00–7:00 when SOA_M_ was the most aged, driven by the relatively higher LWC and SO_4_^2−^ (Figure S1). The average ratio of P/M (1.0 ± 0.8) at the foot of Mt. Huang was comparable to that (0.89) in Hefei, which is nearly 200 km farther away from the sampling site, and was much lower than the ratios of the urban aerosols in North China and South China in summer [5]. Additionally, the 3-HGA/MBTCA ratio can be adopted to investigate the formation pathway of SOA_M_ from *α*-pinene or *β*-pinene, because the oxidation of *α*-pinene relative to *β*-pinene can form more MBTCA than HGA [28]. The HGA/MBTCA ratio is as low as 1.0 when *α*-pinene makes a more significant contribution to SOA_M_ [35,37]. The daytime (1.6 ± 0.9) and night-time (1.7 ± 1.1) ratios of 3-HGA/MBTCA were close to 1.0, indicating that *α*-pinene was a more important precursor of SOA_M_ compared to *β*-pinene at Mt. Huang.

#### 2.2.3. *β*-Caryophyllene SOA Tracers

As one of the predominant sesquiterpenes, *β*-caryophyllene can be emitted from pine trees, sunflower, corn, and other crops [38]. *β*-caryophyllinic acid is generated from the ozonolysis or photochemical oxidation of *β*-caryophyllene, and thus it can be proposed as an indicator for SOA from sesquiterpenes [10]. Interestingly, the pattern of diurnal changes in *β*-caryophyllic acid was consistent with levoglucosan (the tracer of biomass burning) (Table 1 and Table 2), suggesting an important effect of biomass burning on the formation of *β*-caryophyllic acid. Consistent with the pattern of diurnal variations in SOA_I_ and SOA_M_, the *β*-caryophyllinic acid concentration presented a higher concentration in the night-time than that in the daytime (Table 2) and peaked at 4:00–7:00 (Figure 2).

### 2.3. Comparison of BSOA Tracers with Other Sites

**Figure 5:**
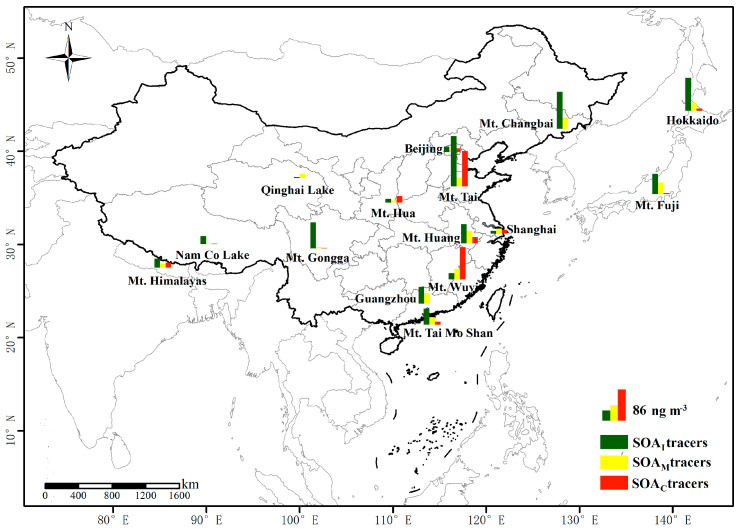
Concentrations (ng m^−3^) of BSOA tracers at Mt. Huang and other areas of the world during the summer (concentrations of SOA_C_ tracers enlarged by ten times for visualization).

A comparison of the concentration level of BSOA tracers over Mt. Huang with other previous studies is shown in Figure 5. The molecular distribution of BSOAs at Mt. Huang followed the order of SOA_I_ > SOA_M_ > SOA_C_, which is consistent with that in other regions, except for Mt. Wuyi and Qinghai Lake where SOA_M_ was the dominant species (Figure 5). PM_2.5_ samples were collected at the foot of Mt. Huang (483 m a. s. l) during the summer in this study, when the sampling site was mainly covered by evergreen and deciduous broad-leaved mixed forests [39]. However, the summit of Mt. Hua (29.8 ng m^−3^) in the North China Plain is covered with little vegetation and most of the land is barren [13]. Thus, the total concentration (112.3 ± 37.8 ng m^−3^) of BSOA tracers at the foot of Mt. Huang was much higher than that at Mt. Hua (29.8 ng m^−3^) in summer. Interestingly, we found that the concentration (66.8 ng m^−3^) of SOA_I_ tracers at Mt. Huang was comparable to that (69.0 ng m^−3^) at Mt. Fuji of Japan [14], primarily because both are dominated by broad-leaved forests. Isoprene is mainly emitted from broad-leaved trees and herbaceous plants [28,32]. The concentration of SOA_I_ tracers in this study was lower than that at the top of Mt. Gongga (88.6 ng m^−3^) [38] and Mt. Tai (93.8 ng m^−3^) [40] and Hokkaido (113 ng m^−3^, a rural site near forest) [41], largely because of the weaker solar radiation at the foot of Mt. Huang. However, the SOA_I_ concentration at Mt. Huang was higher than that in other mountainous regions such as Mt. Tai Mo Shan (54.7 ng m^−3^) [42], Mt. Hua (13.0 ng m^−3^) [13], and Mt. Wuyi (21.0 ng m^−3^) [12], as well as in the continental background regions in the Tibetan Plateau [43], Nam Co Lake (26.6 ng m^−3^), and Qinghai Lake (3.8 ng m^−3^) [32]. As shown in Figure 4, the concentration of SOA_I_ tracers was higher in the eastern regions such as Mt. Tai, Mt. Changbai, and Mt. Wuyi than that in the western regions such as Mt. Hua and Mt. Himalayas, which was related to the different vegetation types dominated by the natural geographical environment characteristics (e.g., climate, precipitation, and solar radiation).

The concentration of SOA_M_ (43.2 ng m^−3^) over Mt. Huang was similar to that in other mountainous regions, such as Mt. Wuyi (36.0 ng m^−3^) [12], Mt. Fuji (39.0 ng m^−3^) [14], and Mt. Changbai (40.0 ng m^−3^) [44]. In addition, this concentration was higher than that in China’s megacities, such as Guangzhou (35.1 ng m^−3^) [10], Shanghai (17.7 ng m^−3^) [45], and Beijing (10.5 ng m^−3^) [46], because Mt. Huang is densely covered by conifers, which can emit large quantities of SOA_M_ [46]. The SOA_C_ concentration (2.2 ng m^−3^) at Mt. Huang was one order of magnitude higher than that at Qinghai Lake (0.09 ng m^−3^) [32] and over Mt. Gongga (0.13 ng m^−3^) [38], but was lower than that in China’s urban areas, such as Beijing (6.88 ng m^−3^) [47], Hefei (6.55 ng m^−3^), and Wuxi (6.73 ng m^−3^) [29].

### 2.4. Effects of Anthropogenic Sources and Meteorological Parameters on BSOA Formation

The correlations between BSOA tracers and temperature, relative humidity, O_3_, pH_is_, LWC, SO_4_^2−^, NO_3_^−^, and levoglucosan are shown in Figure 4. The BSOA tracers derived from isoprene, monoterpene, and *β*-caryophyllene were correlated strongly with O_3_ (*r* = 0.63–0.73, *p* < 0.01) in the daytime, whereas there was no correlation (−0.35 ≤ *r* ≤ −0.33, *p* > 0.05) at night, which was also observed over Mt. Tai [6]. O_3_ and OH· radicals have been considered key atmospheric oxidants during the daytime, while NO_3_ radicals are important oxidants at night [48,49]. Additionally, the chemical sink of O_3_ by the residual NO titration at night may be an alternate reason for the insignificant correlation of BSOA tracers with O_3_ at night [50]. Therefore, it can be concluded that BSOAs at the foot of Mt. Huang during the daytime were primarily generated from the photo-oxidation of BVOCs with O_3_, whereas BSOAs at night may be mainly derived from the oxidation with NO_3_ radicals and other oxidants. Each BSOA tracer showed no correlation with temperature throughout the whole observation period (*r* from −0.13 to +0.20, *p* > 0.05, Figure 4), indicating that the effect of temperature on the production of BSOAs was negligible at Mt. Huang. The increased temperature can accelerate biogenic VOC emissions and further favor the BSOA formation, as reported in previous studies [13,51,52]. Nevertheless, the increase in temperature can promote the volatilization of BSOAs, resulting in the decreased concentration of BSOAs in the aerosol phase [53,54]. The mountain-valley breezes could also be responsible for such insignificant correlations.

SO_4_^2−^ is a representative product of aqueous oxidation, and thus the correlation between SOA tracers and SO_4_^2−^ can be used to investigate the aqueous formation process of BSOAs [32,55]. BSOA tracers exhibited stronger correlations (*r* = 0.66−0.83, *p* < 0.01) with SO_4_^2−^ in the night-time than those (*r* = 0.48–0.49, 0.01 < *p* < 0.05, and *r* = 0.80, *p* < 0.01) in the daytime (Figure 4), indicating the enhanced aqueous formation processes of BSOAs at night. Chamber studies and field measurements have demonstrated that the acidic condition of particles can promote BSOA formation [31,51]. Lin et al. [30] pointed out that acidic aerosols containing sulfate could promote the conversion of IEPOX into 2-methyltetrols and other IEPOX-derived SOA tracers. As shown in Figure 4, each BSOA tracer presented a significant negative correlation (−0.75 ≤ *r ≤* −0.68, *p* < 0.01) with pH_is_ during the daytime and night-time, confirming that the acidic environment of aerosols is beneficial to the BSOA generation through acid-catalyzed heterogeneous oxidation. Field measurements reported that BSOAs exhibited significant negative correlations with RH over Mt. Hua and Mt. Wuyi [12,13], while BSOAs displayed no relationship with LWC over Mt. Hua and Mt. Tai. Moreover, the chamber study reported that the lower RH favored the formation of 2-methylglyceric acid and its corresponding oligomers, whereas the effect of RH on 2-methyltetrols was negligible [33]. In this study, BSOA tracers showed negative correlations with RH (−0.47 ≤ *r* ≤ −0.35, *p* < 0.05) during the night-time and moderate correlations with LWC (*r* = 0.37- 0.48, *p* < 0.05) (Figure 4). High RH and LWC concentration could enhance the gas-to-particle partitioning of volatile organic precursors of BSOAs into the aqueous phase, and ultimately facilitate the BSOA formation. However, high RH and LWC concentration could inhibit the BSOA formation via acid-catalyzed oxidation due to the dilution of particle acidity. Additionally, BSOA tracers showed stronger correlations with RH and LWC during the night-time than those during the daytime (Figure 4).

Previous studies pointed out that anthropogenic pollutants could promote BSOA formation [12,55]. SO_4_^2−^ originates from the aqueous oxidation of SO_2_ emitted from the combustion of petroleum and coal [56], and thus it can be proposed as an important indicator for anthropogenic pollutants. There were positive correlations between BSOA tracers and SO_4_^2−^ in the day and night as discussed above, indicating the enhancing effect of SO_4_^2−^ on BSOA generation. One reason for the significant influence of SO_4_^2−^ on BSOAs was that an enhancement of SO_4_^2−^ could promote the ring-opening reaction of IEPOX and further SOA formation [32,57]. In addition, the salting-in effect of SO_4_^2−^ could enhance the solubility of polar organic compounds such as IEPOX [45,55]. These results demonstrated that the higher concentration of SO_4_^2−^ can augment SOAs by promoting gas-particle conversion and aqueous-phase oxidation [27]. Nevertheless, there were moderate correlations (*r* = 0.40–0.59, *p* < 0.05) between BSOA tracers and NO_3_^−^, primarily because of the volatilization of NO_3_^−^ during long-distance transport [58]. Levoglucosan is principally derived from the pyrolysis of cellulose and hemicellulose at temperatures higher than 300 °C, and thus it can be recognized as a significant tracer of biomass burning [25]. There were moderate or strong correlations of levoglucosan with BSOA tracers (*r* = 0.55–0.78, *p* < 0.05, Figure 4), suggesting that biomass burning from long-distance transport exerted a significant effect on BSOAs. Because of its lower volatile property, *β*-caryophyllene is abundant in leaves and wood. It is emitted to the atmosphere only through biomass burning [59]. Thus, levoglucosan exhibited stronger correlations (*r* = 0.78, *p* < 0.01) with SOA_C_ tracers than SOA_I_ and SOA_M_ (with *r* = 0.55, *p* < 0.05, or *r* = 0.61–0.75, *p* < 0.01), indicating that biomass burning exerted a more significant effect on SOA_C_ than SOA_I_ and SOA_M_. Such a phenomenon was consistent with our previous observation in Liaocheng on the North China Plain [60].

### 2.5. Source Apportionment

To assess the relative contributions of primary and secondary sources of organic aerosols, the SOA tracer method can be used to estimate their relative abundances in *OC*. In this study, the tracer yield method proposed by Kleindienst et al. [52] was applied to estimate the contribution of secondary organic carbon (*SOC*) derived from the oxidation of isoprene, monoterpene, and *β*-caryophyllene. The *f_SOC_* (conversion coefficient of BSOA tracers) values were reported to be 0.155 ± 0.039, 0.231 ± 0.111, and 0.023 ± 0.005 for isoprene, monoterpenes, and *β*-caryophyllene, respectively. According to Zhang et al. [61] and Zhang et al. [62], the contribution of biomass burning was calculated via the concentration of levoglucosan, and the *f_SOC_* was 0.080 ± 0.033. It is noteworthy that the SOA tracer method possesses a certain degree of uncertainties, as discussed by El Haddad et al. [63] and Yttri et al. [64] in detail. However, such an assessment was considered the most scientific available method thus far to provide meaningful insights into the diurnal trends of SOA tracers. The equation for calculating [*SOC*]*_i_* is given as follows:

(1) $${\left[SOC\right]}_{i}=\sum_{i}\left[tri\right]/f_{SOC}$$

where [*SOC*]*_i_* is the concentration of *SOC* generated by a specific VOC precursor *i*; Σ*_i_*[*tri*] is the sum of the concentrations of tracers derived from precursor *i* oxidation. Equations used in the calculation of *OC* contributions are illustrated as follows:

(2) $$Contribution\;of\;{BSOA}_{I}\;tracers=\left(C_{2-MTs}+C_{C5-\mathrm{alkane}}+C_{2-MG}\right)/\left(0.155\times OC\right)\times 100\%$$

(3) $$Contribution\;of\;{BSOA}_{M}\;tracers=\sum {BSOA}_{M}/\left(0.231\times OC\right)\times 100\%$$

(4) $$Contribution\;of\;{BSOA}_{C}\;tracers=C_{caryophyllinic}/\left(0.023\times OC\right)\times 100\%$$

(5) $$Contribution\;of\;biomass\;burning=C_{levoglucosan}/\left(0.080\times OC\right)\times 100\%$$

In the above equations, *C*_2*-MTs*_, *C_C_*_5_*_-_*_alkane_, *C*_2-*MG*_, *C_caryophyllinic_*, and *C_levoglucosan_* refer to the concentrations of 2-methyltetrols, *C*_5_-alkene triols, 2-methylglyceric acid, *β*-caryophyllinic acid, and levoglucosan in aerosols, respectively; ∑*_BSOA__M_* refers to the sum of *cis*-pinonic acid, *cis*-pinic acid, 3-hydroxyglutaric acid, and 3-methyl-1,2,3-butanetricarboxylic acid.

**Figure 6:**
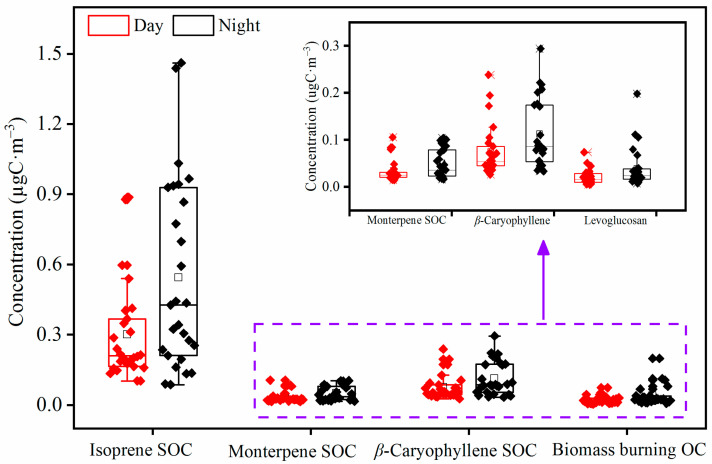
Diurnal variations in secondary organic carbon (SOC) from biogenic sources and organic carbon (OC) from biomass burning in PM_2.5_ samples of Mt. Huang.

The calculated OCs are shown in Table 2 and Figure 6. The total calculated OC mass concentrations were 0.55 ± 0.11 µgC·m^−3^ and 0.93 ± 0.23 µgC·m^−3^ during the day and night, accounting for 12.7 ± 6.0% and 15.1 ± 8.4% of the sum of OC, respectively (Table 2). The diurnal variations in primary OC and biogenic SOC were consistent with the concentration levels of the detected organic species characterized by the higher night-time values than in daytime. The concentration of biogenic SOC (0.71 ± 0.40 µgC·m^−3^) was more than 20 times higher than that (0.03 ± 0.03 µgC·m^−3^) of primary OC (biomass-burning-derived OC). Isoprene-derived SOC was the most abundant species during the daytime and night-time, followed by monoterpene-, *β*-caryophyllene-derived SOC, and biomass-burning-derived OC. These four species accounted for 6.8 ± 2.8%, 3.6 ± 1.4%, 1.8 ± 0.9%, and 0.5 ± 0.5% of OC during the daytime and 8.8 ± 5.6%, 3.7 ± 2.1%, 2.0 ± 1.4%, and 0.6 ± 0.4% during the night-time, respectively.

The average concentration of biomass-burning-derived OC was 0.03 ± 0.03 µgC·m^−3^, which was equal to that (0.03 ± 0.01µgC·m^−3^) over Mt. Fuji. However, the contribution of biomass-burning-derived OC to total OC concentrations was only 0.6 ± 0.5% during the entire sampling period, which was much less than that (24%) in early June over Mt. Tai where organic aerosols were significantly influenced by biomass burning, and smaller than that (2.3%) over Mt. Fuji of Japan where the effect of biomass burning was minor, indicating the negligible influence of biomass burning on organic aerosols at the foot of Mt. Huang. The relative abundance (13.4 ± 6.9%) of estimated biogenic SOC in aerosol OC at Mt. Huang was almost the same as that in the urban aerosol of Jinan (13.3%) [65] and higher than that over Mt. Tai in June (5.5%) [26] and a rural site (5.5%) in Shanxi Province of China [9], but lower than at Mt. Fuji (37.2%) [14], Mt. Gongga (16.6%) [38], and a rural site in the central Pearl River Delta (38.4%) [51] in the summer.

## 3. Materials and Methods

### 3.1. Sample Collection

Mt. Huang (30.1° N, 118.2° E, 1840 m a. s. l) is situated in the southwest of the Yangtze River Delta (YRD), East China. Climate in this area is heavily regulated by the East Asian monsoon with a marked seasonal variability in temperature, cloudiness, and relative humidity. More than 90% of the mountainous land is covered by vegetation, which comprises broad-leaved forests at low altitudes and coniferous forests at high altitudes. The climate of Mt. Huang varies significantly on a daily basis, owing to the contrasting topography of high mountains and deep valleys.

The monitoring site is located at the Anhui Academy of Forestry of Mt. Huang (30.1°N, 118.2°E, 483 m a. s. l, Figure 3), which is situated at the foot of Mt. Huang. There are not any obvious anthropogenic emissions near the sampling site, and thus it can reflect the air quality of alpine background sites in East China. The PM_2.5_ sampling at a 3 h time resolution was conducted from July 27 to August 3 in 2019 using a high-volume air sampler (TE-6070-BLX, TISCH, New York, NY, USA) coupled with prebaked (450 °C for 6 h) quartz fiber filters at an airflow rate of 1.13 m^−3^ min^−1^. Two blank samples were collected at the beginning and the end of the sampling period by installing the filters onto the sampler for about 15 min without pumping any air. A total of 53 PM_2.5_ samples and 2 field blank samples were collected in this study. After sampling, each filter was sealed in an aluminum bag and stored at −20 °C prior to analysis.

Gaseous pollutants (e.g., CO, SO_2_, NO_2_, and O_3_) and meteorological parameters including temperature, relative humidity, and wind speed/direction at the foot of Mt. Huang were retrieved from the website of Chinese Air Quality Monitoring System (http://www.aqistudy.cn, accessed on 8 September 2022) at a one-hour time resolution. Planetary boundary layer (PBL) height was obtained from the website of Copernicus Meteorological Center (https://cds.climate.copernicus.eu/cdsapp#!/dataset/reanalysis-era5-single-levels?tab, accessed on 15 October 2022).

### 3.2. Sample Analyses

#### 3.2.1. Organic Compounds

Detailed methods for extraction, derivatization, and gas chromatography/mass spectrometry (GC/MS) analysis have been reported in our previous studies [40,60]. Half of a circle (a diameter of 90 mm) of each filter was cut into pieces and put into a sample bottle. Methylene chloride and a methanol mixed solution (2:1, *v*/*v*) were added and ultrasonic extraction was carried out three times (each time for 15 min). The extracts were filtered through glass wool in a pasteurized dropper and transferred to a pear-shaped bottle; they were then concentrated by a rotary evaporator in vacuum state and dried with pure nitrogen gas. After reacting with a 60 µL mixture of *N*,*O*-bis-(trimethylsilyl)trifluoroacetamide (BSTFA) and pyridine (5:1, *v*/*v*) at 70 °C for 3 h, the derivatives were diluted with 40 µL n-hexane containing the internal standard (C_13_ *n*-hexane) before GC/MS analysis. The derivatized fraction was determined by GC/MS (Agilent, 7890A–5977C, Santa Clara, CA, USA) equipped with a fused silica capillary column (HP-5MS, Santa Clara, CA, USA).

A total of thirteen species of BSOA tracers and levoglucosan were determined (Table 2), including isoprene-derived SOA (SOA_I_) tracers such as two 3-MeTHF-3,4-diols (*trans*-3-methyltetrahydrofuran-3,4-diol and *cis*-3-methyltetrahydrofuran-3,4-diol), three C_5_-alkene triols (*cis*-2-methy-1,3,4-trihydroxy-1-buteme, 3-methy-2,3,4-trihydroxy-1-butene, and *trans*-2-methy-1,3,4-trihydoxy-1-butene), 2-methylglyceric acid, two 2-methyltetrols (2-methylthreitol and 2-methylerythritol), monoterpene-derived SOA (SOA_M_) tracers (e.g., *cis*-pinonic acid, *cis*-pinic acid, 3-hydroxyglutaric acid (3-HGA), and 3-methyl-1,2,3-butanetricarboxylic acid (MBTCA)), and one *β*-caryophyllene-derived SOA (SOA_C_) tracer (*β*-caryophyllenic acid).

#### 3.2.2. Organic Carbon (OC), Elemental Carbon (EC), and Inorganic Ions

OC and EC were analyzed using a DRI Model 2015 Carbon Analyzer following the Interagency Monitoring of Protected Visual Environments (IMPROVE) thermal/optical reflectance (TOR) protocol. A 0.5026 cm^2^ filter membrane was placed in the analyzer sample boat and the PM_2.5_ samples were heated sequentially to 140 °C (OC1), 280 °C (OC2), 480 °C (OC3), and 580 °C (OC4) in an oxygen-free pure helium (HE) environment. The temperature was gradually increased to 580 °C (EC1), 740 °C (EC2), and 840 °C (EC3) under the condition of He containing 2% oxygen. The limits of detection for OC and EC are 0.08 μg·m^−3^ and 0.06 μg·m^−3^, respectively.

Inorganic ions were analyzed by ion chromatography (Dionex-Aquion, Dionex-600, Thermo Fisher, Waltham, MA, USA). An aliquot of each sample filter was extracted with 30 mL of Milli-Q water using an ultrasonic bath three times and then filtered through PTFE filters to remove particles and filter debris, and finally identified using by ion chromatography.

#### 3.2.3. Quality Assurance and Quality Control

Field blanks were extracted and measured in the same way as the PM_2.5_ samples, and no obvious target compounds (less than 3%) were found in the field blanks. The recovery experiment was performed by spiking the standard solution onto blank filters and recoveries of the target compounds ranged from 85% to 120%. Method detection limits for 2-methylthreitols, 3-methyl-2,3,4-trihydroxy1-butene, 2-methylglyceric acid, 3-HGA, and *β*-caryophyllinic acid are 0.04, 0.05, 0.08, 0.05, and 0.12 ng m^−3^, respectively.

### 3.3. Aerosol Liquid Water Content and Particle In Situ pH

In this study, liquid water content (LWC) and particle in situ pH (pH_is_) in the PM_2.5_ samples were calculated using the ISORROPIA-II model. This model treats Na^+^-NH_4_^+^-K^+^-Ca^2+^-Mg^2+^-SO_4_^2−^-NO_3_^−^-Cl^−^ as a system [27]. The forward and metastable mode in the ISORROPIA model was employed.

### 3.4. Backward Trajectories

Air mass backward trajectories arriving at Mt. Huang were calculated using the Hybrid Single-Particle Lagrangian Integrated Trajectory Model (HYSPLIT model, http://ready.arl.noaa.gov/HYSPLITraj.php, accessed on 16 September 2022). Meteorological data were retrieved from NOAA air resources laboratory (http://www.arl.gov/ready/hysplit.html, accessed on 16 September 2022). These trajectories were run eight times every day (02:00, 05:00, 08:00, 11:00, 14:00, 17:00, 20:00, and 23:00 UTC) with the arrival level at 1000 m. The method of the trajectories used the GIS-based software TrajStat 1.5.4 [66].

## 4. Conclusions

The diurnal variations in BSOA tracers derived from isoprene, monoterpene, and *β*-caryophyllene were analyzed for PM_2.5_ samples collected at the foot of Mt. Huang during the summer. We found that SOA_I_ tracers were the most abundant species, accounting for 56.1% and 61.5% of the total BSOA tracers during the day and night, followed by SOA_M_ tracers (41.9% and 36.7%) and SOA_C_ tracers (2.1% and 1.9%). The night-time concentrations of levoglucosan, OC, EC, and SIA were also higher at night than during the day. Nearly all of the BSOA tracers exhibited higher concentrations at night than in the day. In addition, the cumulative effect of the dynamic transport of atmospheric pollutants through the mountain breeze at night was another crucial reason for the higher BSOA tracers. There was a positive correlation between BSOA tracers and O_3_ during the day rather than at night, indicating that BSOA tracers were primarily generated from the photo-oxidation of BVOCs with O_3_ only during the day. BSOA tracers were strongly correlated with pH_is_ and SO_4_^2−^, indicating that BSOAs were produced from the acid-catalyzed aqueous-phase oxidation. The robust correlation of levoglucosan with BSOA tracers, especially *β*-caryophyllinic acid, suggested that biomass burning from long-distance transport exerted a significant effect on BSOA formation.

## Figures and Tables

**Table 1:** Meteorological parameters and concentrations (µg m^−3^) of major chemical compositions in PM_2.5_ samples at the foot of Mt. Huang during the summer.

	Daytime (*n* = 26)	Nighttime (*n* = 27)	Total (*n* = 53)
I. Meteorological parameters and gaseous pollutants			
T (°C)	33.0 ± 3.7	30.3 ± 2.7	31.7 ± 3.5
RH (%)	64.9 ± 14.9	75.6 ± 12.2	70.3 ± 14.6
Wind speed (m·s^−1^)	2.0 ± 1.2	1.5 ± 0.6	1.7 ± 0.7
PBL (m)	923.1 ± 467.1	301.0 ± 106.5	612.1 ± 457.5
O_3_ (ppb)	72.8 ± 10.2	51.7 ± 12.4	62.3 ± 17.4
NO_2_ (µg m^−3^)	9.9 ± 1.5	10.1 ± 1.4	9.9 ± 1.7
SO_2_ (µg m^−3^)	7.2 ± 2.7	10.2 ± 1.0	8.8 ± 2.5
II. Inorganic ions (µg m^−3^)			
SO_4_^2−^	1.4 ± 1.0	2.2 ± 1.4	1.8 ± 1.3
NO_3_^−^	0.4 ± 0.2	0.5 ± 0.3	0.5 ± 0.2
NH_4_^+^	0.7 ± 0.4	0.8 ± 0.7	0.8 ± 0.6
SIA	2.5 ± 1.2	3.5 ± 1.9	3.0 ± 1.6
III. Other species			
OC (µg m^−3^)	4.9 ± 3.1	7.2 ± 4.9	6.1 ± 4.3
EC (µg m^−3^)	0.4 ± 0.3	1.0 ± 0.7	0.7 ± 0.4
OC/EC	17.7 ± 11.9	18.4 ± 12.5	18.1 ± 12.2
* SOC (µg m^−3^)	2.4 ± 1.3	6.2 ± 3.3	4.3 ± 3.3
pH_is_	5.4 ± 2.4	4.4 ± 2.1	4.9 ± 2.3
LWC (µg m^−3^)	3.1 ± 3.0	4.0 ± 3.8	3.6 ± 3.5
Levoglucosan (ng m^−3^)	1.6 ± 1.3	3.2 ± 3.3	2.4 ± 2.6
PM_2.5_ (µg m^−3^)	12.3 ± 6.3	14.5 ± 8.1	13.3 ± 7.3

* SOC = OC − (OC/EC)_min_ × EC.

**Table 2:** Diurnal variations in biogenic organic aerosol (BSOA) concentrations, primary OC, and biogenic SOC concentrations and contributions.

	Daytime (*n* = 26)	Night-time (*n* = 27)	Total (*n* = 53)
I. Isoprene-derived SOA (ng m^−3^)			
3-MeTHF-3,4-diols	3.0 ± 1.2	4.1 ± 1.9	3.5 ± 1.7
2-Methylglyceric acid	1.4 ± 1.5	1.2 ± 0.9	1.3 ± 1.2
C_5_-alkene triols	18.6 ± 18.1	47.6 ± 39.6	33.1 ± 32.9
2-Methyltetrols	23.6 ± 13.5	34.1 ± 23.6	28.8 ± 19.9
Subtotal	46.6 ± 32.2	87.0 ± 61.2	66.8 ± 52.9
II. Monoterpene-derived SOA (ng m^−3^)			
*cis*-Pinic acid	3.2 ± 0.2	3.3 ± 0.3	3.2 ± 0.2
*cis*-Pinonic acid	4.8 ± 0.3	4.8 ± 0.2	4.8 ± 0.2
3-Hydroxyglutaric acid	13.9 ± 10.0	22.1 ± 14.0	17.8 ± 12.8
MBTCA	13.0 ± 12.7	21.7 ± 18.7	17.3 ± 16.3
Subtotal	34.8 ± 24.1	51.9 ± 30.8	43.2 ± 28.9
III*. β*-Caryophyllene-derived SOA (ng m^−3^)			
*β*-Caryophyllinic acid	1.7 ± 1.2	2.6 ± 1.6	2.2 ± 1.5
Total BSOA tracers	83.1 ± 54.8	141.6 ± 89.4	112.3 ± 37.8
Ratios			
P/M	1.1 ± 0.6	0.8 ± 0.6	1.0 ± 0.8
3-HGA/MBTCA	1.6 ± 0.9	1.7 ± 1.1	1.7 ± 1.1
BSOA/OC (%)	1.9 ± 0.8	2.3 ± 1.3	2.1 ± 1.1
IV. Estimated OC mass concentration (µgC·m^−3^)			
Isoprene SOC	0.30 ± 0.21	0.56 ± 0.39	0.43 ± 0.34
*α*-Pinene SOC	0.15 ± 0.10	0.22 ± 0.13	0.19 ± 0.12
*β*-Caryophyllene SOC	0.08 ± 0.05	0.11 ± 0.07	0.09 ± 0.06
Biomass burning OC	0.02 ± 0.01	0.04 ± 0.03	0.03 ± 0.03
Sum of biogenic SOC	0.53 ± 0.34	0.90 ± 0.57	0.71 ± 0.40
Subtotal	0.55 ± 0.11	0.93 ± 0.23	0.74 ± 0.18
VI. Percentage in OC (%)			
Isoprene SOC	6.8 ± 2.8	8.8 ± 5.8	7.8 ± 4.4
*α*-Pinene SOC	3.6 ± 1.7	3.7 ± 2.1	3.7 ± 1.9
*β*-Caryophyllene SOC	1.8 ± 0.9	2.0 ± 1.4	1.9 ± 1.2
Biomass burning OC	0.5 ± 0.5	0.6 ± 0.4	0.6 ± 0.5
Sum of biogenic SOC	12.2 ± 5.0	14.5 ± 8.5	13.4 ± 6.9
Subtotal	12.7 ± 6.0	15.1 ± 8.4	13.9 ± 4.3

## Data Availability

The data for this paper will be made available on request from the corresponding author.

## Supplementary Materials

The following supporting information can be downloaded at: https://www.mdpi.com/article/10.3390/molecules28165939/s1, Figure S1: Diurnal variations of temperature (T), relative humidity (RH), and the concentrations of SO_4_^2−^, NO^3−^, liquid water content (LWC), and in situ particle pH (pH_is_) in PM_2.5_. Figure S2. Linear correlations of 3-MeTHF-3,4-diol with C_5_-alkene triols.
