# Supplementary material for: Diurnal Variations in High Time-Resolved Molecular Distributions and Formation Mechanisms of Biogenic Secondary Organic Aerosols at Mt. Huang, East China
Source: Molecules. 2023 Aug 8;28(16):5939. doi: 10.3390/molecules28165939 (PMC10458846; doi:10.3390/molecules28165939)
Supplement: Supplementary file 1 [file molecules-28-05939-s001.zip › molecules-2488177-supplementary.pdf]

Supplementary data

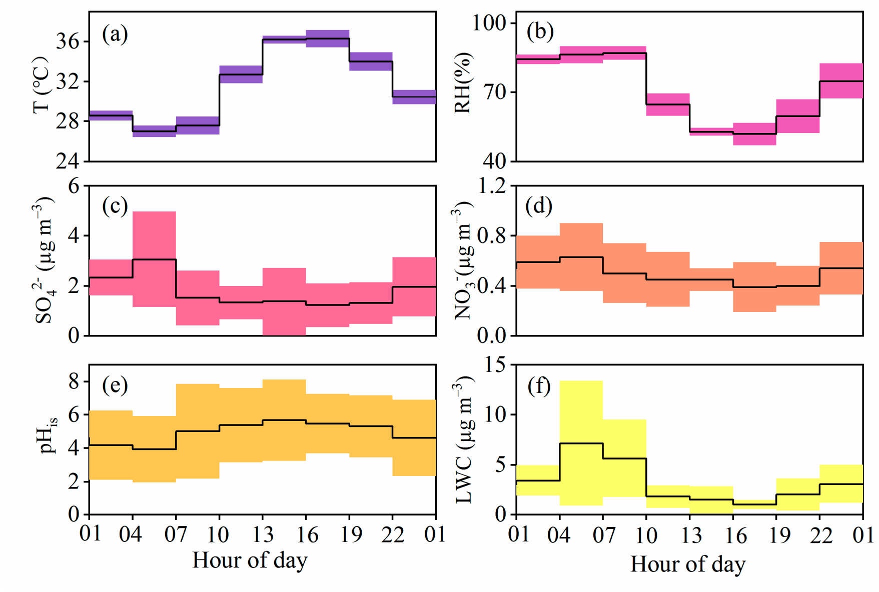

Figure S1. Diurnal variations of temperature (T), relative humidity (RH), and the concentrations of SO_4^{2-}, NO_3^-, liquid water content (LWC), and *in situ* particle pH (pH_{is}) in $PM_{2.5}$.

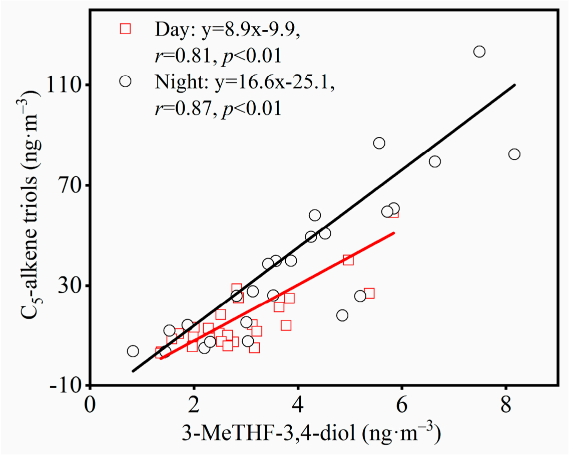

Figure S2. Linear correlations of 3-MeTHF-3,4-diol with C_5-alkene triols.
